# Evidence-Based Intrapartum Care: A Retrospective Descriptive Assessment of Facility-Based Births in Rural Public Health Facilities in Midwestern Uganda

**DOI:** 10.7759/cureus.89581

**Published:** 2025-08-07

**Authors:** Brian Turigye, Joseph Ngonzi, Jonathan Kajjimu, Arnold Kamugisha, Edgar M Mulogo

**Affiliations:** 1 Department of Community Health, Mbarara University of Science and Technology, Mbarara, UGA; 2 Department of Obstetrics and Gynecology, Mbarara University of Science and Technology, Mbarara, UGA; 3 Department of Emergency Medicine, Mbarara University of Science and Technology, Mbarara, UGA

**Keywords:** evidence-based practice, intrapartum care, maternal-child health, quality of care, uganda

## Abstract

Introduction

Efforts to reduce maternal and newborn deaths, especially in sub-Saharan Africa, have not been sufficient to achieve Sustainable Development Goal (SDG) 3 for 2030. The quality of care around childbirth is critical for both mothers and newborns, and the use of evidence-based practices (EBPs) is vital in ensuring optimal outcomes. However, there is a paucity of recent research on the use of evidence-based practices in childbirth health facilities.

Aims

This study aimed to describe the use of intrapartum evidence-based practices for facility-based births in rural lower public health facilities in Midwestern Uganda.

Methods

A descriptive retrospective chart review was conducted of mothers who delivered at all public health facilities in the Kasese and Bundibugyo districts. Mothers who had given birth in 42 health facilities two months preceding the study period were systematically sampled using probability proportionate to the size of expected respective facility births. Data was collected between November and December 2024. A structured questionnaire adopted from the World Health Organization (WHO) Quality Maternal and Newborn Care (QMNC) was used to extract data on the use of EBPs. A descriptive analysis was done, and the results were presented as frequencies and percentages.

Results

Of the 353 mothers, 73.1% (n=258) were monitored using a partograph. Blood pressure (BP) was the least monitored at only 23.6% (n=83). At admission, apart from Leopold’s examination, which was performed and recorded in 86.7% (n=306) of the charts, all other practices were below 50%. About 32.3% (n=114) of the charts had a documented management plan, and all expected laboratory investigations were in less than 2% of the charts. The record of the examination of newborns prior to discharge was low, at 15.6% (n=55). The coverage of EBPs was lowest in hospitals and highest in facilities at the health center 3 (HCIII) level.

Conclusions

The findings indicate a need for strategies and research to improve the implementation of evidence-based practices in rural public health facilities, especially in hospitals.

## Introduction

Improving maternal and newborn outcomes remains a global priority [1]. One of the key goals, as set in Sustainable Development Goal (SDG) 3, is to reduce maternal mortality rates to less than 70 per 100,000 live births and neonatal deaths to less than two per 1,000 live births by 2030 [2]. However, this progress has been slowed [3]. The global estimates indicate about 260,000 maternal deaths [4] and 2.3 million newborn deaths every year [5]. Just over 90% of these deaths are in low-income and lower-middle-income countries and are due to preventable causes [4]. Improving the quality of maternal and newborn care, especially around childbirth, has been shown as one of the priority interventions to avert these maternal and newborn deaths [6].

To accelerate the reduction in maternal and newborn deaths, in 2016, the Uganda Ministry of Health (MOH) implemented the Reproductive, Maternal, Newborn, Child, and Adolescent Health (RMNCAH) Sharpened Plan [7]. There has since been an exponential increase in health facility births from 76.6% in 2016 to 88% in 2022 [8,9]. While there has been a reduction in the maternal mortality ratio from 336 per 100,000 live births in 2016 to 189 in 2022 and neonatal deaths from 27 per 1,000 live births in 2016 to 22 per 1,000 in 2022, the figures are still far above the SGD and national targets of 2030 [9,10]. Evidence now suggests that increased facility births, without improving the quality of care, do not necessarily reduce maternal and newborn deaths [11]. The coverage of quality care for facility-based births in Uganda’s health facilities has been previously reported to be low [12]. Hence, improving the quality of care during childbirth is of significant importance to reduce these maternal and newborn deaths.

The use of evidence-based practices (EBPs) is an important approach in improving the quality of care within health facilities [13]. EBPs are developed from high-quality research to guide best clinical practices. Evidence indicates that incorporating these EBPs into clinical practice during childbirth improves both maternal and newborn outcomes [14]. Implementing intrapartum evidence-based practices (EBPs) in clinical care requires maternity care providers to be equipped with the knowledge and skills on World Health Organization (WHO)-recommended intrapartum guidelines [14]. The World Health Organization (WHO) incorporated the EBPs into the WHO Quality Maternal and Newborn Care (QMNC) framework for quality improvement and to optimize maternal and newborn outcomes [15]. However, the use of these key practices remains low globally [16]. Nationwide estimates in Uganda also show a generally low coverage of maternal and newborn interventions during childbirth at mainly lower public health facilities in rural regions, such as Midwestern Uganda [12]. Despite the integration of the WHO EBPS within Uganda’s healthcare practice, there is limited regional-level research on their use during childbirth in Midwestern Uganda, since most studies have been done in purposively selected facilities. Additionally, studies that have evaluated some of these evidence-based practices have concentrated on tertiary hospitals, yet the majority of childbirths in Uganda take place in rural lower-level public health facilities [12].

This study therefore aimed to describe the use of intrapartum evidence-based practices for facility-based births in rural lower public health facilities in Midwestern Uganda.

## Materials and methods

Study design

This was a descriptive retrospective chart review conducted between November and December 2024.

Study setting

The study was conducted in the Kasese and Bundibugyo districts, in the Midwestern Region of Uganda. Maternal and newborn care in the two districts is delivered through district-level public, private-for-profit, and Private-not-for-profit facilities. This study was conducted in only public health facilities since there were challenges with getting administrative clearance from other facilities. The district-level health facilities are categorized into health center 2 (HCII), health center 3 (HCIII), health center 4 (HCIV), and district hospital. However, care during childbirth is provided from HCIII and above, with HCII supporting family planning services, antenatal care (ANC), and postnatal care (PNC) services. We therefore only conducted our studies in HCIII, HCIV, and hospital-level facilities. The two districts are served by 43 public health facilities, i.e., two hospitals, four HCIVs, and 37 HCIIIs, but one of the HCIIIs was not offering childbirth services at the time and was excluded. We therefore recruited participants from 42 public health facilities that were offering childbirth care services in the two districts.

Study participants

We utilized charts of all mothers who delivered in the 42 public health facilities in the Kasese and Bundibugyo districts two months before the survey (between November and December 2024). A sample size of 350 mothers using a single population formula in Epi Info (CDC, Atlanta, GA) was calculated. A 36% level of use of evidence-based practices from a previous study was used as the proportion (p) at a 95% confidence interval and 5% margin of error [17]. Probability proportionate to size was used to determine the sample size for each health facility based on the expected number of monthly deliveries from the district health office registry. We set a minimum number of five mothers from each facility for facilities that had very few expected deliveries. Systematic random sampling was used to select mothers for chart review from these health facilities. We used the calculated sample size for each facility to determine the sampling fraction (k). Once the study team reached the facility, all the charts for mothers who delivered in the study period were retrieved from the facility in charge. The kth number was calculated as the total number of available eligible mothers’ charts in the facility divided by the sample size allocated to the health facility. The index participant was selected using a lottery method and systematically selected other charts until the sample size was achieved.

Data collection

Data was collected by two clinical research assistants with a bachelor’s degree in medicine and surgery, supported by one midwife who was trained on the study procedures for five days. On average, two facilities were enrolled per day. A de-identified questionnaire was used to extract information from the mothers’ charts by the research assistants. The questionnaire was adopted from the WHO QMNC framework and modified for the Uganda setting [15]. It was also based on previous related studies [17]. A panel of experts (two midwives, two obstetricians, and two maternal and newborn public health specialists) was used to review the list of questions, the relevance of response options, clarity, and appropriateness to assess the intrapartum use of evidence-based care. Extracted information in the questionnaire included the mother’s social demographic information, admission notes, physical examination, partograph, delivery information, and discharge information. The details of EBPs assessed in the questionnaire and their response options are available in the Appendices. The questionnaire is also available in the Appendices. Data was securely entered and stored using the REDCap software version 12.2.11 (Vanderbilt University, Nashville, TN).

Data analysis

Extracted data was exported from REDCap and analyzed using the Stata software version 18.5 (StataCorp LLC, College Station, TX). Descriptive statistics were used to show the characteristics of the participants and the assessed intrapartum care practice items. For each item, a response code of either “1” for yes or “0” for no was assigned, based on whether the item was performed and recorded or not. Items with missing values or any response other than “yes,” such as not recorded to any of the questions, were considered as “no.” Results were presented as frequencies with percentages and medians with interquartile ranges (IQRs) for continuous variables. Fisher’s exact test was used to check for any differences across the levels of facilities. A P value of p<0.05 was used as the level of statistical significance.

Ethical consideration

This study was conducted based on international and national standards, as guided by the Uganda National Council for Science and Technology (UNCST). Ethical approval was granted by the Mbarara University of Science and Technology (MUST) Research Ethics Committee (MUST-2024-1726) and the UNCST (HS5213ES). Additional administrative clearance was obtained from the district health offices of Kasese and Bundibugyo to carry out the study in the selected health facilities. Since this study used records of mothers, consent was waived by the ethics committee. These charts were de-identified while entering data into REDCap.

## Results

Characteristics of the study participants

Of the 353 mothers enrolled in the study, 266 (75.4%) were from Kasese, while 87 (24.6%) were from Bundibugyo. The majority, 269 (76.2%), gave birth from HCIII, and 181 (51.3%) were multiparous. The median duration of time between admission and delivery was four hours (interquartile range {IQR}: 2-7), while the median duration of stay in the facility was 27 hours (IQR: 24-46 hours) (Table 1).

**Table 1:** Characteristics of the study participants (N=353) HC, health center; IQR, interquartile range

Variable	Frequency, n (%)
District	
Bundibugyo	87 (24.6%)
Kasese	266 (75.4%)
Health facility level	
HCIII	269 (76.2%)
HCIV	50 (14.2%)
Hospital	34 (9.6%)
Gestational age at delivery (weeks), median (IQR)	38 (37-38)
Parity	
Primipara	91 (25.8%)
Multipara	181 (51.3%)
Grand multipara	81 (22.9%)
Referred in	8 (2.3%)
Total duration of time between admission and delivery (hours), median (IQR)	4 (2-7)
Total duration of stay in the health center (hours), median (IQR)	27 (24-46)
Fetal condition at admission	
Dead	2 (0.6%)
Alive	347 (98.3%)
Not recorded	4 (1.1%)

Performance and documentation of physical examinations, laboratory investigations, and management plans at admission

The majority of the mothers, 306 (86.7%), had Leopold’s examination performed, and 264 (74.8%) had a documented human immunodeficiency virus (HIV) test. However, only 128 (36.6%) had a documented temperature, 43.8% (154) had a documented pulse rate, and 168 (47.7%) had a documented blood pressure (BP). There was a significant difference in the performance of these examinations across the different levels of facilities, with the lowest performance being in hospitals. In the laboratory investigations, no mother was screened for syphilis; only one (0.3%) had a complete blood count (CBC) on file, and four (1.1%) had a urinalysis. Only 114 (32.3%) of the mothers had a documented management plan at admission, and this varied significantly across facility levels, with the lowest documentation (70, 26.0%) at HCIII and the highest at the hospital (20, 58.8%) (Table 2).

**Table 2:** Vitals taken and examination done and recorded at admission †P values were generated using Fisher’s exact test due to low cell counts (with more than 20% of cells having expected counts below five or any cell below one) *P<0.05, which was the selected significance level HIV, human immunodeficiency virus; ANC, antenatal care; HC, health center

Variable	Health facility level				
	HCIII, n=269	HCIV, n=50	Hospital, n=34	Total, n=353	P value†
Blood pressure (BP) taken	137 (50.9%)	30 (61.2%)	1 (2.9%)	168 (47.7%)	<0.001*
Pulse rate (PR) taken	122 (45.5%)	31 (62.0%)	1 (2.9%)	154 (43.8%)	<0.001*
Temperature	112 (42.1%)	16 (32.0%)	0 (0.0%)	128 (36.6%)	<0.001*
Leopold’s examination	234 (87.0%)	45 (90.0%)	27 (79.4%)	306 (86.7%)	0.358
Admission fetal heartbeat	150 (56.4%)	24 (49.0%)	13 (38.2%)	187 (53.6%)	0.106
Documented management plan at admission	70 (26.0%)	24 (48.0%)	20 (58.8%)	114 (32.3%)	<0.001*
Urinalysis	1 (0.4%)	3 (6.0%)	0 (0.0%)	4 (1.1%)	0.002*
Complete blood count (CBC)	0 (0.0%)	1 (2.0%)	0 (0.0%)	1 (0.3%)	0.048*
Blood group and rhesus antigen	0 (0.0%)	3 (6.0%)	0 (0.0%)	3 (0.8%)	<0.001*
Syphilis screening	0 (0.0%)	0 (0.0%)	0 (0.0%)	0 (0.0%)	-
HIV testing either at ANC or delivery and test results documented	219 (81.4%)	34 (68.0%)	11 (32.4%)	264 (74.8%)	<0.001*

Use of the partograph for monitoring labor

The majority of mothers, 258 (73.1%), were monitored using a partograph. This usage varied significantly across facility levels, with the highest partograph use at health center 3 (237, 88.1%) and only three (8.8%) at the hospital level. Nearly all mothers, 336 (95.2%), had their delivery times recorded, while antenatal care (ANC) information was the least documented, in only 55 (15.6%). Additionally, 58.9% of the partographs included all 30-minute fetal heart rate recordings, 216 (61.2%) recorded contractions every 30 minutes, 170 (48.2%) documented liquor assessments every four hours, 207 (58.6%) included four-hourly cervical dilation recordings, and 196 (55.5%) noted descent every four hours. However, less than a quarter of the partographs (83, 23.6%) had blood pressure measured and recorded every four hours (Table 3).

**Table 3:** Documentation of fetal vital information, labor progress, and maternal monitoring using a partograph †P values were generated using Fisher’s exact test due to low cell counts (with more than 20% of cells having expected counts below five or any cell below one) *P<0.05, which was the selected significance level ANC, antenatal care; HC, health center

Variable	Health facility level				
	HCIII, n=269	HCIV, n=50	Hospital, n=34 (9.6%)	Total, n=353	P value†
Mothers’ vital information recorded					
a) Admission time	258 (95.9%)	44 (88.0%)	24 (70.6%)	326 (92.4%)	<0.001*
b) Delivery time	264 (98.1%)	44 (88.0%)	28 (82.4%)	336 (95.2%)	<0.001*
c) Gestational period	255 (94.8%)	43 (86.0%)	28 (84.8%)	326 (92.6%)	0.019*
d) ANC information	28 (10.4%)	14 (28.0%)	13 (38.2%)	55 (15.6%)	<0.001*
e) Time of the onset of labor	175 (65.1%)	18 (36.0%)	17 (50.0%)	210 (59.5%)	<0.001*
f) Time of rupture of the membrane	158 (58.7%)	10 (20.0%)	1 (2.9%)	169 (47.9%)	<0.001*
Partograph used to monitor labor	237 (88.1%)	18 (36.0%)	3 (8.8%)	258 (73.1%)	<0.001*
Fetal heart rate (every 30 minutes)					
Not recorded	38 (14.1%)	26 (52.0%)	27 (79.4%)	91 (25.8%)	<0.001*
Some recorded	38 (14.1%)	9 (18.0%)	7 (20.6%)	54 (15.3%)	
All recorded	193 (71.7%)	15 (30.0%)	0 (0.0%)	208 (58.9%)	
Contractions (every 30 minutes)					
Not recorded	33 (12.3%)	30 (60.0%)	30 (88.2%)	93 (26.3%)	<0.001*
Some recorded	35 (13.0%)	5 (10.0%)	4 (11.8%)	44 (12.5%)	
All recorded	201 (74.7%)	15 (30.0%)	0 (0.0%)	216 (61.2%)	
Liquor (every four hours)					
Not recorded	38 (14.1%)	34 (68.0%)	32 (94.1%)	104 (29.5%)	<0.001*
Some recorded	76 (28.3%)	1 (2.0%)	2 (5.9%)	79 (22.4%)	
All recorded	155 (57.6%)	15 (30.0%)	0 (0.0%)	170 (48.2%)	
Cervical dilation (every four hours)					
Not recorded	28 (10.4%)	26 (52.0%)	27 (79.4%)	81 (22.9%)	<0.001*
Some recorded	49 (18.2%)	9 (18.0%)	7 (20.6%)	65 (18.4%)	
All recorded	192 (71.4%)	15 (30.0%)	0 (0.0%)	207 (58.6%)	
Descent (every four hours)					
Not recorded	54 (20.1%)	30 (60.0%)	30 (88.2%)	114 (32.3%)	<0.001*
Some recorded	34 (12.6%)	5 (10.0%)	4 (11.8%)	43 (12.2%)	
All recorded	181 (67.3%)	15 (30.0%)	0 (0.0%)	196 (55.5%)	
Blood pressure (every four hours)					
Not recorded	97 (36.2%)	33 (66.0%)	33 (97.1%)	163 (46.3%)	<0.001*
Some recorded	94 (35.1%)	11 (22.0%)	1 (2.9%)	106 (30.1%)	
All recorded	77 (28.7%)	6 (12.0%)	0 (0.0%)	83 (23.6%)	
Pulse rate (every four hours)					
Not recorded	103 (38.3%)	37 (74.0%)	34 (100.0%)	174 (49.3%)	<0.001*
Some recorded	60 (22.3%)	8 (16.0%)	0 (0.0%)	68 (19.3%)	
All recorded	106 (39.4%)	5 (10.0%)	0 (0.0%)	111 (31.4%)	
Temperature (every four hours)					
Not recorded	132 (49.1%)	41 (82.0%)	33 (100.0%)	206 (58.5%)	<0.001*
Some recorded	65 (24.2%)	4 (8.0%)	0 (0.0%)	69 (19.6%)	
All recorded	72 (26.8%)	5 (10.0%)	0 (0.0%)	77 (21.9%)	
Documented use of the active management of the third stage of labor applied and documented	257 (97.3%)	44 (88.0%)	32 (94.1%)	333 (95.7%)	0.010*
Pain control was done and documented	156 (58.4%)	33 (66.0%)	8 (24.2%)	197 (56.3%)	<0.001*

Immediate postpartum newborn care

The majority of births, 311 (88.1%), were delivered by spontaneous vaginal delivery and 39 (11.0%) by Caesarean section, while there was no birth by assisted vaginal delivery. Most of the charts (299, 84.7%) of the delivery notes had a documented skin-to-skin contact, 294 (83.8%) had a documented initiation of breastfeeding within an hour of birth, and 281 (79.8%) had a documented receipt of vitamin K injection. Only 24 (6.8%) had a complete record of newborn vital signs, and 55 (15.6%) had a record of complete newborn examination before discharge (Table 4).

**Table 4:** Performance and documentation of best practices in newborn care †P values were generated using Fisher’s exact test due to low cell counts (with more than 20% of cells having expected counts below five or any cell below one) *P<0.05, which was the selected significance level TTC, tetracycline; HC, health center

Variable	Health facility level				
	HCIII, n=269	HCIV, n=50	Hospital, n=34	Total, n=353	P value†
Skin-to-skin contact immediately after birth	240 (89.2%)	34 (68.0%)	25 (73.5%)	299 (84.7%)	<0.001*
Early initiation of breastfeeding (within one hour after birth)	235 (87.7%)	35 (70.0%)	24 (72.7%)	294 (83.8%)	0.002*
Are there record of vital signs of the newborn?					
Not recorded	194 (72.1%)	43 (86.0%)	33 (97.1%)	270 (76.5%)	0.009*
Some recorded	53 (19.7%)	5 (10.0%)	1 (2.9%)	59 (16.7%)	
All recorded	22 (8.2%)	2 (4.0%)	0 (0.0%)	24 (6.8%)	
Does the record show the complete examination of the newborn before discharge?	50 (18.6%)	5 (10.0%)	0 (0.0%)	55 (15.6%)	0.010*
Newborn provided vitamin K and documented	227 (84.4%)	37 (75.5%)	17 (50.0%)	281 (79.8%)	<0.001*
Newborn provided TTC eye ointment and documented	219 (81.4%)	37 (75.5%)	21 (61.8%)	277 (78.7%)	<0.001*
Type of delivery					
Spontaneous vaginal delivery	262 (97.4%)	23 (46.0%)	26 (76.5%)	311 (88.1%)	<0.001*
Caesarean section	4 (1.5%)	27 (54.0%)	8 (23.5%)	39 (11.0%)	
Not documented	3 (1.1%)	0 (0.0%)	0 (0.0%)	3 (0.8%)	
Assisted delivery type	0 (0.0%)	0 (0.0%)	0 (0.0%)	0 (0.0%)	

## Discussion

The present study set out to examine the use of intrapartum evidence-based practices in rural public health facilities in Midwestern Uganda. Generally, there were marked facility-level differences in the quality of intrapartum care provided, where HCIIIs, despite being lower-level facilities, outperformed both HCIVs and hospitals. We found that overall, almost three-quarters of the mothers are monitored using partographs but with key partograph features being inconsistently recorded. This level of partograph usage is higher than previously reported elsewhere in Uganda [18-20]. However, there was a significant difference in usage across the different facility levels. The highest usage was in HCIII, with more than 80% of mothers monitored using a partograph, while the lowest was in the hospital, with less than 10% mothers of labor. These findings are similar to previous studies that have found very low partograph in hospitals [18]. This disproportionately low use of partographs in hospitals highlights a need for targeted support at these facilities. One of the reasons that has been attributed to this is high staff workload amidst high patient volume in hospitals, which leaves midwives with difficulties in performing the evidence-based practices according to standard [21-23]. There is therefore an urgent need to boost staffing levels, especially in hospitals.

Despite the high level of partograph use, nearly half of the partographs did not have a complete record of fetal heart rate, contractions, liquor, cervical dilation, and descent. BPs were the least monitored, with less than a quarter having complete BP records. Notably, even at admission, less than a quarter of the mothers had a BP record. These findings are consistent with previous studies done in Northern Uganda [19,20]. This is concerning since hypertensive disorders remain one of the leading causes of maternal mortality globally and in Uganda [10,24]. At admission, the majority of the mothers had a documented Leopold’s examination. However, less than half of the mothers did not have a record of fetal heart rate, pulse rate, and temperature. These findings are similar to studies done in Central and Northern Uganda [18,20]. Alongside work overload, the low readiness of these facilities, such as the lack of equipment such as blood pressure machines and knowledge gaps, could explain the lack of proper BP monitoring [12,20]. The proper monitoring of vitals such as BPs and fetal heart rate is very crucial for the early identification of danger signs and proper management. It is therefore important that more efforts such as support supervision are made toward improving these practices [20,25]. Additionally, the poor monitoring of labor observed in our setting may also be attributed to the lack of adequate training of health providers and the inherent complexity in using partographs to monitor labor in resource-constrained environments [26]. But it is interesting to see interventions such as intrapartum care algorithms, which have been developed to serve as decision aids or reminders for enhancing the utilization of WHO’s recommended intrapartum EBPs, especially in low-resource settings [27,28]. There is also a need to strengthen the facilities’ in-house continuing medical education (CMEs) to focus regularly on such gaps.

Overall, newborn care practices were well documented, particularly skin-to-skin contact immediately after birth, the early initiation of breastfeeding within the first hour, the receipt of tetracycline eye ointment, and a vitamin K injection. These are in agreement with similar studies that also noted the highly effective utilization of neonatal care EBPs [14,29]. However, these findings are contrary to those from a recent nationwide study that found the low coverage of postpartum practices [12].

However, the vital sign monitoring and examination of the newborn were very low after delivery, with less than 10% of the charts having a record of a newborn vital sign and less than a quarter showing a complete examination of the newborn before discharge. This is consistent with a previous analysis of the Uganda demographic health survey that found the low coverage of immediate postnatal care checks [30]. This is concerning and highlights the need for a root cause analysis. Factors such as healthcare provider motivation, attitudes, and commitment to routine newborn care might need to be further investigated.

Among nearly all mothers (95.7%), the active management of the third stage of labor, including the administration of uterotonics immediately after birth, was done and documented. This is consistent with findings of similar studies done in developing countries such as Sri Lanka [14].

Despite ongoing efforts by our Ministry of Health to advocate for intrapartum EBPs, there remains a need for increased attention, capacity building, and supervision across all facility levels, particularly in rural areas.

Strengths and limitations

This study surveyed all the public health facilities that delivered childbirth care in the Kasese district, hence giving a true representation of care for facility-based birth in the area. It, however, had some limitations. First, we used mothers’ charts to gather data. We therefore had no opportunity to clarify missing information. Additionally, some of the practices could have been performed but not documented. Our study, however, was also very interested in proper documentation on practices, and the use of charts was relevant. Also, our study only included mothers who gave birth in public health facilities.

The assessment of evidence-based practices (EBPs) in this study relied primarily on patient charts, which are known to be poorly documented in our setting. Moreover, several EBPs, such as encouraging women to adopt birthing positions of choice, ensuring privacy, allowing companionship during labor, and promoting women’s mobility during labor, may be practiced but are often not captured in routine documentation. The direct observation of care and interviews with women or healthcare providers would have provided a more accurate assessment of the actual implementation of EBPs.

Our study was limited to public health facilities, which may not fully represent the broader landscape of EBP utilization in the study setting. Future research should consider comparing public with private facilities to identify more comprehensive implementation challenges and strategies across the full spectrum of maternity care in the country.

## Conclusions

The findings show that there is a generally high level of partograph use at lower-level facilities, although it is very low in hospitals. Also, generally, the performance of EBPs was lower in hospitals. There is therefore a need for strategies to improve the use of evidence-based practices in these hospitals. There were also low-level physical examinations, laboratory investigations, and vital sign monitoring, which are crucial in proper management. There is a need for more research focused on barriers to providing evidence-based practices, especially in tertiary hospitals, which would provide data to guide future programs on ensuring the use of EBPs.

## Appendices

Table 5 shows the details of EBPs assessed in the questionnaire and their response options.

**Table 5:** Summary of evidence-based practices assessed TTC, tetracycline; ANC, antenatal care; HIV, human immunodeficiency virus; WHO, World Health Organization; QMNC, Quality Maternal and Newborn Care

Evidence-based practices assessed		
Admission	Standard QMNC item checked	Response options
Physical examination	Blood pressure	Yes, no, and not recorded
	Temperature	Yes, no, and not recorded
	Leopold’s examination	Yes, no, and not recorded
	Admission fetal heartbeat	Yes, no, and not recorded
	Pulse rate (PR)	Yes, no, and not recorded
	Documented management plan at admission	Yes, no, and not recorded
Laboratory test	Urinalysis	Yes, no, and not recorded
	Complete blood count (CBC)	Yes, no, and not recorded
	Blood group and rhesus antigen	Yes, no, and not recorded
	Syphilis screening	Yes, no, and not recorded
	HIV testing either at ANC or delivery and test results documented	Yes, no, and not recorded
Partograph	Mothers’ vital information recorded	Response options
	Admission time	Recorded and not recorded
	Delivery time	Recorded and not recorded
	Gestational period	Recorded and not recorded
	ANC information	Recorded and not recorded
	Time of the onset of labor	Recorded and not recorded
	Time of rupture membrane	Recorded and not recorded
Partograph used to monitor labor		Response options
	Fetal heart rate (every 30 minutes)	Not recorded, some recorded, and all recorded
	Contractions (every 30 minutes)	Not recorded, some recorded, and all recorded
	Liquor (every four hours)	Not recorded, some recorded, and all recorded
	Cervical dilation (every four hours)	Not recorded, some recorded, and all recorded
	Descent (every four hours)	Not recorded, some recorded, and all recorded
	Blood pressure (every four hours)	Not recorded, some recorded, and all recorded
	Pulse rate (every four hours)	Not recorded, some recorded, and all recorded
	Temperature (every four hours)	Not recorded, some recorded, and all recorded
	The partograph was plotted correctly (use the WHO standard for partograph plotting)	Not recorded, some recorded, and all recorded
	The partograph plotting of the progress of labor was used for making timely decisions	Not recorded, some recorded, and all recorded
	Documented use of the active management of the third stage of labor applied and documented	Not recorded, some recorded, and all recorded
	Pain control was done and documented	Not recorded, some recorded, and all recorded
Newborn care practices	Standard QMNC practice	Response options
	Skin-to-skin contact immediately after birth	Yes, no, and not recorded
	Early initiation of breastfeeding (within one hour after birth)	Yes, no, and not recorded
	Vital signs of the newborn	Yes, no, and not recorded
	Complete examination of the newborn before discharge	Yes, no, and not recorded
	Newborn provided with vitamin K and documented	Yes, no, and not recorded
	Newborn provided TTC eye ointment and documented	Yes, no, and not recorded

Table 6 shows the questionnaire.

**Table 6:** Questionnaire LNMP, last menstrual period; MTCT, mother-to-child transmission; ARV, antiretroviral; TTC, tetracycline; ANC, antenatal care; HIV, human immunodeficiency virus; WHO, World Health Organization; CBC, complete blood count; Rh, rhesus; VDRL, venereal disease research laboratory; BEmONC, basic emergency obstetric and newborn care

Section 1: sociodemographic and obstetric variables		
1	Interview code	
2	Was the patient referred?	
3	Place of referral	
4	Place of residence	
5	Level of education	
6	Occupation	
7	Gestational age at delivery (calculate if LNMP is specified) and maternal age	Weeks: 77, unknown; 99, not recorded. Years: 77, unknown; 99, not recorded
8	Parity	99, not recorded
9	Admission diagnosis	99, not recorded
10	Stage of labor at admission	No labor. Latent first stage. Active first stage. Second stage; 99: not recorded
11	Any complication diagnosed at admission	No complication detected. Hypertension in pregnancy. Bleeding/hemorrhage. Prolonged/obstructed labor. Premature rupture of the membrane. Preterm labor. Fetal distress. Others (specify); 99: Not recorded
12	Total duration of time between admission and delivery	Hours: 99, not recorded
13	Total duration of stay in the health center	Hours: 99, not recorded
14	Fetal condition at admission	0, dead; 1, alive; 99, not recorded

Section 2: review of clinical care at admission		
1	All necessary vital signs are taken at admission and documented properly	Blood pressure (BP): yes; no. Pulse rate (PR): yes; no. Temperature: yes; no; 99, not documented
2	Physical examination is done at admission and documented	Leopold’s examination: yes; no. Pelvic examination: yes; no. Fetal heartbeat: yes; no; 99, not documented
3	At admission, there is a documented management plan	Not documented; documented but not complete; documented and complete
4	Basic laboratory tests done and documented at admission	Urine albumin: yes; no. CBC: yes; no. Blood group and Rh: yes; no. VDRL: yes; no; 99, no documented
5	HIV testing was done at ANC or delivery, and test results are documented	No; yes; 99, not documented
6	What was the test result of the mother?	Nonreactive; reactive; not documented
7	If the woman was reactive, were she and the newborn provided ARV prophylaxis to prevent MTCT?	No; yes; 99, not documented

Section 3: recording of follow-up care. Please observe the presence of and note the following details on the partograph labor care guide or chart		
1. Does the chart have each of the following client information?		
a) Admission time	0. No	1. Yes
b) Delivery time	0. No	1. Yes
c) Gestational period	0. No	1. Yes
d) ANC information	0. No	1. Yes
e) Onset of labor	0. No	1. Yes
f) Time of rupture membrane	0. No	1. Yes

2. Observe the frequency of entries made in relation to time when the observations are made for the following parameters. Tick whether all entries are made			
a) Fetal heart rate (every 30 minutes)	0. Not recorded	1. Some recorded	2. All recorded
b) Contractions (every 30 minutes)	0. Not recorded	1. Some recorded	2. All recorded
c) Liquor (every four hours)	0. Not recorded	1. Some recorded	2. All recorded
d) Cervical dilation (every four hours)	0. Not recorded	1. Some recorded	2. All recorded
e) Descent (every four hours)	0. Not recorded	1. Some recorded	2. All recorded
f) Blood pressure (every four hours)	0. Not recorded	1. Some recorded	2. All recorded
g) Pulse rate (every four hours)	0. Not recorded	1. Some recorded	2. All recorded
h) Temperature (every four hours)	0. Not recorded	1. Some recorded	2. All recorded
3. Was a partograph used to follow the progress of labor?	0. No; 1. Yes	-	-
4. Was the partograph plotted correctly (use the WHO standard for partograph plotting or BEmONC training manual)?	0. No; 1. Yes		
5. Was the partograph plotting of the progress of labor used for making timely decisions on the management of the progress of labor?	0. No; 1. Yes		
6. How do you rate the use of the partograph (plotting and use for decision)?	Very bad. Bad. Fair. Good. Very good		
7. Active management of the third stage of labor applied and documented	0. No; 1. Yes; 99. Not documented		
8. Pain control was done and documented	0. No; 1. Yes; 99. Not documented		
9. Was there any maternal or fetal/neonatal complication detected at any stage of the labor and delivery before discharge?	1. No maternal or fetal complication detected. Section 4 hypertension in pregnancy or preeclampsia. Bleeding/postpartum hemorrhage. Prolonged/obstructed labor. Premature rupture of the membrane. Postpartum infection. Preterm labor. Fetal distress. Neonatal infection. Low-birth-weight baby. Others (specify); 99: not documented		
10. If the woman had any of the maternal complications in Q9, was the mother managed in line with the WHO guidelines?	0. No; 1. Yes; 99. Not documented		
11. If the newborn had any of the complications in Q9 (fetal distress, neonatal infection, or low birth weight baby), was the newborn managed in line with the WHO guideline (refer to the guideline)	0. No; 1. Yes		

Section 4: newborn care	
1. Sex of child	Male; female; 99, not documented
2. Weight of child	____ kg; 99, not documented
3. Apgar score of the newborn	First minute and fifth minute; 99, not documented
4. Skin-to-skin contact immediately after birth	0. No; 1. Yes; 99. Not documented
5. Time to cord clamping (delayed cord clamping)	____ seconds; 99, not documented
6. Early initiation of breastfeeding (within one hour after birth)	0. No; 1. Yes; 99. Not documented
7. Are there records of vital signs of the newborn (pulse rate, temperature, and respiratory rate)?	Not recorded; some recorded; all recorded
8. Does the record show a complete examination of the newborn before discharge?	0. No; 1. Yes
9. Newborn provided with vitamin K and documented	0. No; 1. Yes; 99. Not documented
10. Newborn provided with TTC eye ointment and documented	0. No; 1. Yes; 88. Documented; 99. Not documented

Section 5: outcome of care	
1. Type of delivery	Spontaneous vaginal delivery; assisted delivery (forceps; vacuum); cesarean section; 99, not documented
2. Type of childbirth	Singleton; twin/multiplex
3. What was the maternal outcome of clinical care?	Maternal death; no major complication; developed obstetric complication and managed at the same facility; developed obstetric complication and referred to another facility; unknown/not documented
4. What was the perinatal/fetal outcome of care?	Dead (fresh; macerated); alive; referred to other facility for complication; not documented
